# Long-Term Heat Stress and Genetic Responses in Growth Traits of Thai Native Synthetic Chicken Lines

**DOI:** 10.3390/ani15142130

**Published:** 2025-07-18

**Authors:** Wuttigrai Boonkum, Supawan Wiangnak, Vibuntita Chankitisakul

**Affiliations:** 1 Department of Animal Science, Faculty of Agriculture, Khon Kean University, Khon Kean 40002, Thailand; supawanwiangnak@gmail.com (S.W.); vibuch@kku.ac.th (V.C.); 2 Network Center for Animal Breeding and Omics Research, Khon Kaen University, Khon Kaen 40002, Thailand

**Keywords:** average daily gain, body weight, breast circumference, genetic parameters, genetic trend, heat tolerance, synthetic chicken lines, temperature–humidity index

## Abstract

In this study, we evaluated genetic parameters and progress in growth traits and heat tolerance across four Thai native synthetic chicken lines—Kaimook e-san, Soi Pet, Soi Nin, and Kaen Thong—over seven generations. Growth traits, including body weight (BW), average daily gain (ADG), and breast circumference (BrC), were recorded and analyzed from hatching to 14 weeks of age, with heat stress assessed based on the temperature–humidity index (THI). Genetic parameters were estimated using a multi-trait animal model incorporating THI thresholds. Significant differences in growth traits were observed among the lines (*p* < 0.05). Kaimook e-san consistently exhibited superior performance, with males outperforming females, while Kaen Thong showed the lowest performance across all traits. Heritability estimates declined with age, with higher values observed in early growth stages, indicating stronger genetic influence during early development. Strong negative genetic correlations between heat tolerance and growth traits were observed in Kaimook e-san, while Kaen Thong showed weaker correlations, suggesting greater potential for simultaneous improvement in growth and heat tolerance. All lines demonstrated genetic progress in growth traits across generations, with Kaimook e-san achieving the highest gains, although these were accompanied by a decline in heat tolerance. The findings highlight the trade-offs between growth and thermal resilience and underscore the importance of multi-trait selection strategies for sustainable poultry breeding in tropical environments.

## Simple Summary

In tropical regions, poultry are frequently exposed to elevated ambient temperatures and humidity, which can negatively impact growth performance and survival rates. In this study, we aimed to identify chicken lines that demonstrate both superior growth and enhanced heat tolerance, thereby supporting informed selection for climate-resilient poultry production. By evaluating these traits across multiple generations, we highlight the importance of balancing productivity with environmental adaptability. These insights are vital for shaping future breeding strategies that prioritize not only robust growth but also physiological stability under thermal stress—an increasingly urgent goal in the face of global climate change and the pursuit of sustainable food systems.

## 1. Introduction

Heat stress is a major environmental constraint to poultry production, particularly in tropical regions characterized by persistently high ambient temperatures and humidity [1,2,3]. Although native chickens are generally well adapted to local environments, they remain vulnerable to the adverse effects of prolonged thermal stress, which compromises physiological functions, reproductive performance, and overall productivity [2,4,5,6]. Recent studies have demonstrated that chronic exposure to elevated temperature and humidity detrimentally affects growth performance, hematological parameters, and metabolic stability [7,8].

Heat stress induces physiological responses such as increased respiratory rate, reduced feed intake, and elevated water consumption. Although these mechanisms facilitate thermoregulation, they often compromise nutrient intake and energy balance, ultimately impairing body weight (BW) gain [9,10]. Several studies have consistently reported considerable reductions in BW among heat-stressed chickens compared with those maintained under thermoneutral conditions [11,12]. These challenges are further exacerbated in tropical and resource-limited environments, where access to climate control or mitigation technologies is limited. Despite their partial adaptation, native chickens often exhibit only moderate heat tolerance under sustained or extreme conditions, highlighting the need for genetic and management interventions. Selective breeding for heat-resilient genotypes, integrated with nutritional optimization and improved housing strategies, offers a promising avenue for enhancing heat tolerance without compromising growth traits. Addressing the impacts of heat stress in native poultry is essential not only for sustaining productivity but also for safeguarding food security and supporting rural livelihoods in tropical regions. Understanding the genetic and physiological mechanisms underlying heat resilience remains essential for informing the development of economically viable, context-specific management strategies adapted to tropical production systems.

Although purebred native chickens are well adapted to extreme climatic conditions, their growth performance remains suboptimal [1,13,14], limiting their competitiveness in modern poultry production. Genetic improvement programs targeting purebred native chickens have achieved limited success in enhancing growth traits [15,16]. As a result, the four Thai native chicken synthetic lines used in this study—referred to as Kaimook e-san, Soi Pet, Soi Nin, and Kaen Thong—were developed as part of a long-term genetic improvement program initiated by the Native Chicken Genetic Resource Conservation Project at Khon Kaen University and supported by the Thailand Research Fund. Detailed descriptions of these four synthetic lines were provided by Prowatee et al. [17]. Each line was established through controlled crossbreeding between local native chicken ecotypes (specifically the Thai native Shee breed) and commercial broilers (comprising 50% Thai native and 50% commercial broiler genetics), followed by within-line selection over seven generations. The breeding program aimed to improve specific traits related to production performance, including growth rate, egg production, and heat tolerance. All lines were managed under similar husbandry practices and maintained as closed populations using pedigree-based selection strategies. Selection indices were developed based on estimated breeding values (EBVs) for the targeted traits, and inbreeding was controlled through the implementation of rotational mating designs [17,18,19,20]. Synthetic strains combine the heat tolerance of local chickens and the growth performance of commercial chickens through heterosis. For Thai native synthetic lines, developed through structured selection programs, possess distinct genetic backgrounds that may confer varying degrees of heat resilience. Understanding the genetic consequences of long-term heat exposure on economically important traits is essential for optimizing selection strategies that enhance both productivity and adaptability. In particular, long-term heat stress may alter growth trajectories, feed efficiency, and skeletal development, all of which are integral to genetic evaluations [21,22,23,24,25]. However, most prior studies have focused on short-term heat stress exposure (1–3 years), providing limited insights into chronic effects. Prolonged exposure can result in physiological and metabolic imbalances that affect growth and reproductive performance over time.

Among heat-sensitive phenotypic traits, body weight (BW), average daily gain (ADG), and breast circumference (BrC) are widely recognized as key economic indicators in poultry production due to their strong association with market value, carcass yield, and feed efficiency. These traits are particularly informative under heat stress conditions, as they are sensitive to thermal challenges that impair feed intake, nutrient absorption, and metabolic function. Reduced BW and ADG reflect compromised growth performance, while BrC serves as a proxy for breast muscle development—an economically important carcass component. Therefore, monitoring these traits under heat stress offers a practical and biologically relevant approach to assess both production efficiency and stress resilience in poultry [26]. Long-term evaluations of these traits under heat stress are essential for generating accurate genetic parameters and accelerating the development of heat-resilient breeding lines. Therefore, our objective in this study was to investigate the phenotypic and genetic effects of long-term heat stress on growth traits across four Thai native synthetic chicken lines. The results of this study are expected to inform breeding programs aimed at enhancing thermal adaptability while maintaining genetic gain in economically important traits.

## 2. Materials and Methods

The research was conducted at Khon Kaen University and received ethical approval from the Institutional Animal Care and Use Committee (IACUC) in accordance with the Guidelines for the Ethics of Animal Experimentation established by the National Research Council of Thailand (Approval No. IACUC-KKU-103/65; issued on 15 December 2022). The study was carried out at the experimental farm of the Network Center for Animal Breeding and Omics Research, Faculty of Agriculture, Khon Kaen University, Thailand.

### 2.1. Animal Management

A total of 13,632 individuals from four Thai native synthetic chicken lines (4215 Kaimook e-san; 3840 Soi Pet; 3204 Soi Nin; and 2373 Kaen Thong) over seven generations between 2017 and 2024 were used in this study. Pedigree data were constructed by tracing all generations of ancestors and included 16,442 individuals born between 2016 and 2023. Each generation produced an average of 500 chicks per line. Data included BW at hatching, 4, 8, 12, and 14 weeks of age (BW0, BW4, BW8, BW12, and BW14, respectively); ADG during 0–4, 4–8, 8–12, and 12–14 weeks of age (ADG0–4, ADG4–8, ADG8–12, and ADG12–14, respectively); and BrC at 8, 12, and 14 weeks of age (BrC8, BrC12, and BrC14, respectively). After hatching, the chickens were weighed individually, and an identification number was attached to the leg until 4 weeks of age, followed by wing banding to keep their BW records. All chickens were vaccinated for infectious bronchitis, Newcastle disease, and fowl pox in accordance with the chicken vaccination program. Chickens were provided with water and fed ad libitum using standard commercial broiler feed. Feed was divided into two formulas according to the age of the chickens. From hatching to 4 weeks of age, the feed consisted of 21% crude protein (CP) and 3000 kcal metabolizable energy (ME/kg), followed by a grower feed of 19% CP and 2900 kcal ME/kg. This feed was used until the end of the experiment at 14 weeks of age. All chickens were raised in open-air housing. The photoperiod schedule consisted of two stages. The first stage was from hatching to 4 weeks of age with 24 h of light/0 h dark cycle using warming with a 100-watt lamp, whereas the second stage was from after 5–14 weeks of age with natural light.

### 2.2. Data Collection

Climate data were obtained from the meteorological center closest to the chicken farm (3 km distance). The weather information included daily air temperature (Temp) and relative humidity (RH) recorded every 3 h, which were used to calculate the temperature–humidity index (THI) based on the following formula [27]: THI = (1.8 × Temp + 32) − (0.55 − 0.0055 × RH) × (1.8 × Temp − 26), where Temp is the average temperature in degrees Celsius and RH is the average relative humidity as a percentage. The mean THI for the 4 weeks before the weigh-in for individual birds of each age was used to determine the heat stress threshold and assess genetic parameters. For THI calculation, the average THI values on days 1–28, 29–56, 57–84, and 85–98 before the weigh-in were used with BW0, BW4, BW8, BW12, and BW14, respectively. The average THIs of ADG and BrC were computed the same as those of BW. During this period, birds were exposed to ambient temperatures ranging from 17 °C to 44 °C and relative humidity levels between 48.3% and 97.2%, based on recorded from the Khon Kaen Meteorological Station. All data were verified before genetic analysis using the Proc UNIVARIATE procedure by SAS v.9.0 program to examine data distribution, including assessing normality, homogeneity of variance, and checking data outliers (±3 standard deviation was defined as an outlier). Statistical differences were compared by chicken lines and sex using the post hoc test in the generalized linear model for an unbalanced analysis of variance (GLM procedure) using the SAS package. The onset of heat stress on growth characteristics was tested and analyzed using a simple regression analysis method.

### 2.3. Genetic Analysis

The multi-trait animal model was used to estimate the onset of heat stress and genetic parameters. Based on previous studies by Boonkum et al. [1,28], which investigated the influence of heat stress in the same population, this study adopted the onset of heat stress by the THI was set as 76 (THI76). The THI function was created to estimate the decline in BW, ADG, and BrC under heat stress. The multi-trait animal model, THI function, and variance–covariance structure matrix used in this study were defined as follows.

The multi-trait model used was

Y=Xb+Za+e,

where Y is the vector corresponding to the phenotypic values for the BW, ADG, and BrC traits; X and Z are incidence matrices related to fixed and random effects, respectively; b is the vector of fixed effects, including the chicken hatch set, generation, chicken age, and sex which is sex nested within the THI function ([$f\left(THI\right)$]) to model changes in BW, ADG, and BrC in males and females (i.e., the slope of the regression) in response to varying THI values; a is the vector of random additive genetic effects including without ($a_{0}$) and with ($a_{1}$) consideration of heat stress, assumed to be $a\sim N\left(0,A\sigma_{a0}^{2}\right)$ and $a\sim N\left(0,A\sigma_{a1}^{2}\right),$ where A is an additive relationship matrix and $\sigma_{a0}^{2}$ and $\sigma_{a1}^{2}$ are the additive genetic variance without and with consideration of heat stress; and e is the vector of random residual effects assumed to be $e\sim N\left(0,I\sigma_{e}^{2}\right),$ where I is the identity matrix and $\sigma_{e}^{2}$ is the residual variance.

The THI function was calculated as follows:

$$f\left(THI\right)=\left\{\begin{array}{cc} 0, & THI\leq {THI}_{threshold}\;(no\;heat\;stress)\\ THI-{THI}_{threshold}, & THI>{THI}_{threshold}\;(heat\;stress) \end{array}.\right.$$

Variance components were estimated with the average information restricted maximum likelihood (AIREML) algorithm using the AIREMLF90 program [29]. The AIREMLF90 program was run using the default convergence criterion (ε = 1 × 10^−12^), monitoring both the relative and average absolute changes in variance components. The maximum number of iterations was set to 5000 (OPTION maxrounds 5000), with convergence typically achieved within 8–15 rounds. An optional EM-REML phase of 100 rounds was employed to generate prior variance estimates. Standard errors (SE) for heritability estimates were calculated using the SE_covar_function option. The heritability ${(h}^{2})$ of BW, ADG, and BrC under hot and humid climates were estimated using the equation provided as follows:

$$h^{2}=\frac{\sigma_{a0}^{2}+\sigma_{a1}^{2}+{2\sigma }_{a01}}{\sigma_{a0}^{2}+\sigma_{a1}^{2}+{2\sigma }_{a01}{+\sigma }_{e}^{2}}.$$

Genetic correlations $\left(r_{g}\right)$ between the without (representing the animal’s genetic potential for BW, ADG, and BrC under thermoneutral conditions) and with (indicating sensitivity to heat stress, i.e., the change in BW, ADG, and BrC per unit increase in THI above a given threshold) consideration of heat stress were calculated. These values refer to the genetic components of two or more traits. $r_{g}$ measures the extent to which the same genes or sets of genes influence multiple traits simultaneously. The genetic correlations were calculated as follows:

$$r_{g}=\frac{{COV\sigma }_{a0,a1}}{\sqrt{\sigma_{a0}^{2}*\sigma_{a1}^{2}}}.$$

Genetic progress (∆G) in each trait was estimated based on the equation $\frac{\Delta G}{gen}=\frac{\sigma_{A}\cdot h\cdot i}{gen}$, where $\sigma_{A}\;$= the standard deviation of additive gene effect, h = the accuracy of selection, i = the selection intensity (20% per generation) where $i=\frac{\bar{SD}}{\sigma_{p}}$, $\bar{SD}$ the selection differential (difference between the mean of selected individuals and the population mean), and $\sigma_{p}$ is the phenotypic standard deviation [30], and gen = generation (year of birth of chickens). Heat tolerance as the animal-specific regression slope of estimated breeding values (EBVs) on THI, representing the animal’s genetic sensitivity to heat stress.

## 3. Results

### 3.1. Comparison of Growth Traits

**Figure 1:**
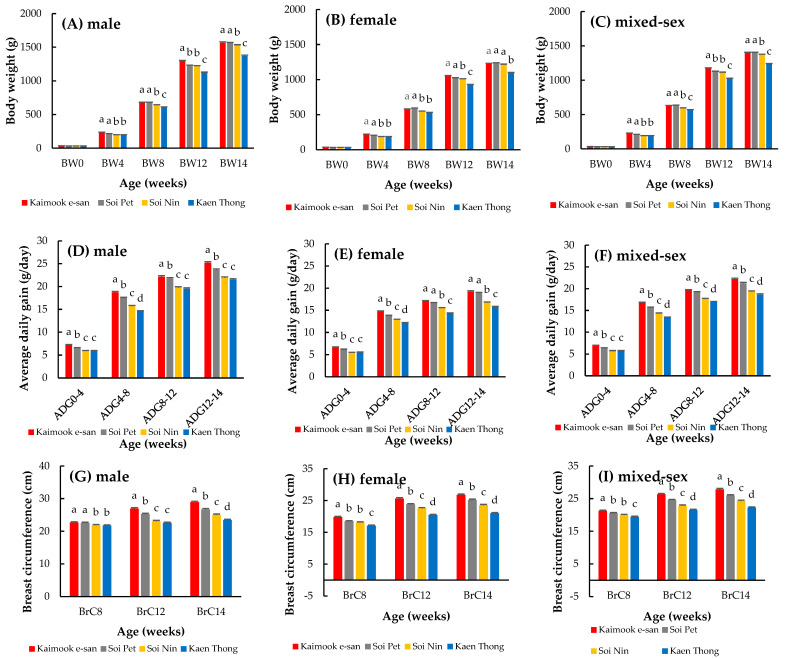
Least-squares means of body weight (BW) (**A**–**C**), average daily gain (ADG) (**D**–**F**), and breast circumference (BrC) (**G**–**I**) across four Thai native synthetic chicken lines and sexes. Different lowercase letters (a, b, c, d) within the same trait indicate statistically significant differences at *p* < 0.05.

The BW, ADG, and BrC of the four Thai native synthetic chicken lines are presented in Figure 1. Across both sexes, Kaimook e-san consistently exhibited the highest BW values, with statistically significant differences from the other lines emerging in BW4 and becoming more pronounced in BW12 and BW14 (*p* < 0.05). By contrast, Soi Pet and Soi Nin maintained intermediate performance levels, whereas Kaen Thong consistently recorded the lowest BW at all time points. ADG followed a similar pattern. Kaimook e-san again demonstrated superior growth efficiency, particularly during ADG8–12 and ADG12–14 (*p* < 0.05), where differences from the other lines were most evident. Soi Pet and Soi Nin remained intermediate, whereas Kaen Thong exhibited the lowest growth rates, particularly in the later growth phases. For BrC, although inter-line differences were less pronounced compared with those for BW and ADG, Kaimook e-san maintained the highest values across all ages and both sexes. By week 14, the BrC in Kaimook e-san males was significantly greater than that in the other lines (*p* < 0.05), underscoring its potential for carcass yield improvement. By contrast, Kaen Thong exhibited the smallest BrC values throughout, reinforcing its characterization as the least growth-oriented line.

### 3.2. Heritability Estimates

The estimated variance components and heritability values for BW, ADG, and BrC across the four Thai native synthetic chicken lines are presented in Table 1. For BW, the heritability estimates (h^2^) were moderate during BW0, ranging from 0.36 ± 0.05 (Kaen Thong) to 0.45 ± 0.05 (Kaimook e-san), indicating a substantial genetic contribution to early growth. However, the h^2^ values declined progressively with age in all lines, reaching their lowest in BW14, from 0.26 ± 0.03 in Kaen Thong to 0.31 ± 0.04 in Kaimook e-san. This trend was accompanied by consistently high additive genetic variances (Va) in later growth phases, particularly in BW14 (ranging from 11,562 to 17,277), reflecting maintained genetic variability in BW. The h^2^ values for ADG followed a similar declining pattern across growth intervals, with the highest values observed during ADG0–4 (0.28–0.34) and the lowest during ADG12–14 (0.22–0.31). Although the magnitude of Va for ADG was relatively moderate (0.75–3.30), the estimates suggest a reasonable potential for early growth selection. The h^2^ values for BrC were also the highest in earlier stages (BrC8: 0.24–0.33) and declined slightly by 14 weeks (BrC14: 0.22–0.28). Additive genetic variances for BrC14 ranged from 0.64 (Kaen Thong) to 1.02 (Kaimook e-san). Overall, these results indicate stronger genetic control during early growth phases and increased environmental influence as birds approach sexual maturity.

### 3.3. Genetic Correlation Estimates

**Figure 2:**
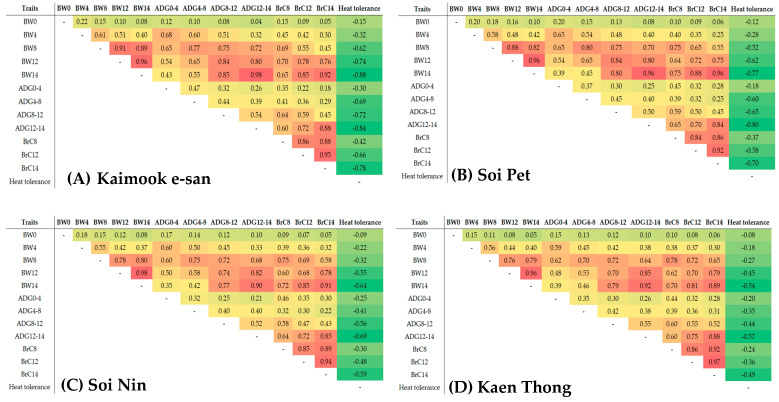
Heatmap of genetic correlations between BW, ADG, BrC, and heat tolerance traits in Kaimook e-san (**A**), Soi Pet (**B**), Soi Nin (**C**), and Kaen Thong (**D**) Thai native synthetic chicken lines. BW0, BW4, BW8, BW12, and BW14 = hatching weight, body weight at 4, 8, 12, and 14 weeks of age, respectively; ADG0–4, ADG4–8, ADG8–12, and ADG12–14 = average daily gain at 0–4, 4–8, 8–12, and 12–14 weeks of age, respectively; BrC8, BrC12, and BrC14 = breast circumference at 8, 12, and 14 weeks of age, respectively.

The heatmap genetic correlations within and between BW, ADG, BrC, and heat tolerance in the four Thai native synthetic chicken lines are presented in Figure 2. All Thai native synthetic chicken lines showed positive genetic correlations within and between BW, ADG, and BrC, with values ranging from 0.04 to 0.98, 0.06 to 0.96, 0.05 to 0.98, and 0.05 to 0.97 for Kaimook e-san, Soi Pet, Soi Nin, and Kaen Thong, respectively. In Kaimook e-san (Figure 2A), heat tolerance traits exhibited consistently strong negative genetic correlations with BW, ADG, and BrC across all age intervals, most notably with BW14 (r = −0.88), ADG12–14 (r = −0.84), and BrC14 (r = −0.78). A similar pattern was observed in Soi Pet (Figure 2B), with genetic correlations between heat tolerance and growth traits ranging from −0.65 to −0.80. The correlation with BrC14 (r = −0.70) further supports a genetic coupling between accelerated growth and vulnerability to heat stress, albeit with slightly attenuated effect sizes relative to those in Kaimook e-san. In Soi Nin (Figure 2C), the negative genetic correlations persisted but were of lower magnitude (r = −0.50 to −0.70), particularly between heat tolerance and BW12 or ADG12–14. Kaen Thong (Figure 2D) exhibited the mildest negative correlations (−0.30 to −0.50). Heat tolerance showed only weak associations with BrC14 and ADG.

### 3.4. Genetic Progress Estimates

The genetic trends per generation (ΔG) for BW, ADG, BrC, and heat tolerance across four Thai native synthetic chicken lines are presented in Table 2. The results showed consistent genetic improvement in growth-related traits, although this progress was accompanied by a concurrent decline in heat tolerance across all lines. Genetic gains in BW from hatching to 14 weeks of age exhibited a positive trajectory, with the highest rates observed in Kaimook e-san. Particularly, Kaimook e-san showed the greatest ΔG for BW at 14 weeks (14.92 g/gen), followed by Soi Pet (13.03 g/gen), Soi Nin (11.18 g/gen), and Kaen Thong (8.10 g/gen). However, these improvements were inversely associated with heat tolerance. Notably, Kaimook e-san and Soi Pet displayed the most substantial genetic declines in heat tolerance at week 14 (−1.49 and −1.26, respectively). Similar patterns were observed in ADG across all growth intervals. The 0–4 week interval exhibited modest gains, with Kaimook e-san again outperforming the other lines (ΔG = 0.54 g/gen), whereas the most pronounced improvements were found during weeks 12–14, where Kaimook e-san and Soi Pet achieved ΔG values of 2.51 and 2.29 g/gen, respectively. By contrast, Kaen Thong consistently exhibited the lowest ADGsacross all intervals. Heat tolerance concurrently declined across all growth periods, with reductions ranging from −0.02 to −0.30, further confirming the antagonistic genetic correlation between accelerated growth and heat tolerance. Genetic progress in BrC was also evident, particularly at 14 weeks. Kaimook e-san again led with the highest ΔG (0.92 cm/gen), whereas Kaen Thong remained the lowest ΔG (0.60 cm/gen). Although the decline in heat tolerance associated with BrC gains followed a consistent trend, the magnitude of reduction was moderate, suggesting that selection for muscling traits may impose a less severe compromise on heat adaptability than selection for BW traits.

**Figure 3:**
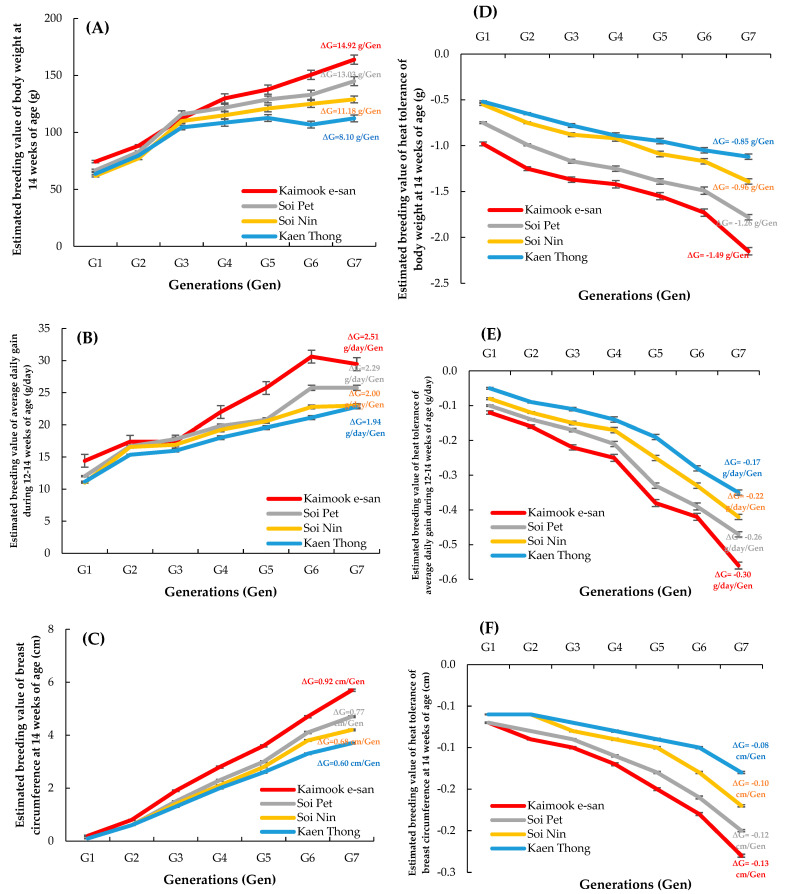
Estimated breeding values (±SE) and genetic progresses (∆G) for BW at 14 weeks of age (**A**), ADG at 12–14 weeks of age (**B**), BrC at 14 weeks of age (**C**), heat tolerance for BW at 14 weeks of age (**D**), ADG at 12–14 weeks of age (**E**), and BrC at 14 weeks of age (**F**) in four Thai native synthetic chicken lines.

The EBVs across seven generations and average genetic progress between growth traits and heat tolerance among the four synthetic chicken lines are presented in Figure 3. In Figure 3A, the EBV for BW14 was the highest in Kaimook e-san, which also demonstrated the greatest average genetic gain (ΔG = 14.92 g/gen), followed by Soi Pet (13.03 g/gen), Soi Nin (11.18 g/gen), and Kaen Thong (8.10 g/gen). As shown in Figure 3B, the EBVs for ADG12–14 demonstrated a consistent upward trend across generations. Kaimook e-san exhibited the highest genetic gain (ΔG = 2.51 g/day/gen), followed closely by Soi Pet (2.29 g/day/gen). The remaining lines, Soi Nin (2.00 g/day/gen) and Kaen Thong (1.94 g/day/gen), showed relatively lower yet stable genetic improvements. Figure 3C illustrates moderate yet consistent increases in EBVs for BrC14. The genetic gain (ΔG) was the highest in Kaimook e-san (0.92 cm/gen) and the lowest in Kaen Thong (0.60 cm/gen), with intermediate improvements observed in Soi Pet (0.77 cm/gen) and Soi Nin (0.68 cm/gen).

Figure 3D–F provide clear visual representations of the antagonistic genetic relationship between growth traits and heat tolerance in the native chicken lines. For BW14, the negative genetic trend in heat tolerance was most pronounced in Kaimook e-san (ΔG = −1.49 g/gen), followed by Soi Pet (−1.26 g/gen), Soi Nin (−0.96 g/gen), and Kaen Thong (−0.85 g/gen). This inverse association was similarly observed for ADG12–14 (Figure 3E), with genetic declines in heat tolerance ranging from −0.30 to −0.17 g/day/gen. Likewise, heat tolerance associated with BrC14 (Figure 3F) showed negative genetic trends, although with smaller magnitudes (−0.13 to −0.08 cm/gen). These findings suggest that selection for structural traits imposes a comparatively lower thermal burden than selection for traits related to metabolic output, reinforcing the need to consider trait-specific thermal sensitivity in breeding programs aimed at improving productivity and resilience.

## 4. Discussion

In this study, we offered a comprehensive evaluation of phenotypic performance, genetic parameters, and long-term genetic trends in four Thai native synthetic chicken lines. The results highlighted substantial genetic gains in BW, ADG, and BrC across seven generations, particularly in Kaimook e-san and Soi Pet lines. These improvements highlighted the effectiveness of directional selection for growth-related traits in native genetic backgrounds. However, the genetic progress in growth traits was consistently accompanied by a measurable decline in breeding values for heat tolerance, indicating a clear antagonistic genetic correlation between heat tolerance and production performance. This inverse relationship aligns with the findings of previous studies reporting trade-offs between metabolic efficiency and environmental resilience in poultry [13,31,32]. The present study is among the few that have examined the genetic response to chronic heat stress over an extended period in indigenous chicken populations. The long-term selection design notably enhanced the reliability of the genetic trends observed and provided valuable insights into the sustainability of current breeding strategies under tropical climatic conditions. These findings emphasize the need to incorporate heat tolerance into multi-trait selection indices to support the development of climate-resilient poultry lines.

Comparative evaluation of growth performance among the four Thai native synthetic chicken lines revealed significant differences in BW, ADG, and BrC, reflecting distinct genetic potentials and selection responses among the lines. These differences were detectable as early as week 4 (BW4) and became more pronounced at later stages (BW12 and BW14), indicating that growth trajectory was strongly influenced by line-specific genetic architecture and selection intensity [33,34]. Kaimook e-san consistently demonstrated superior performance across all growth traits (BW, ADG, and BrC), suggesting effective directional selection for enhanced growth. This finding is consistent with previous studies where sustained selection in native or synthetic populations resulted in measurable improvements in growth traits [18,35,36]. Notably, the strong performance of Kaimook e-san during the 8–12 and 12–14 week intervals suggests a high genetic responsiveness to late-phase growth selection, a critical period for achieving optimal market weight and carcass quality. Sexual dimorphism was clearly observed, with males consistently outperforming females in BW and ADG across all lines. This finding aligns with well-established evidence that sex-linked genetic expression influences growth patterns, primarily due to hormonal differences (e.g., testosterone vs. estrogen) and differential nutrient partitioning [37,38]. Sex-specific differences in growth traits observed in this study may be attributed to underlying physiological and hormonal variations between males and females, which influence their responses to environmental stressors such as heat. Males typically exhibit faster growth rates and higher metabolic heat production, making them more susceptible to heat stress-induced growth suppression. Conversely, females may display greater thermotolerance due to lower metabolic demands and potential differences in endocrine regulation. These sexually dimorphic responses to heat stress are consistent with findings in previous studied [39,40].

At the same time, Kaen Thong exhibited the lowest performance across all traits, which may reflect an adaptive trade-off favoring resilience under heat stress rather than rapid growth. This hypothesis is supported by previous research findings showing that chickens with lower growth rates often display better heat tolerance due to reduced metabolic heat production [41,42]. Therefore, despite its lower productivity than the other lines, Kaen Thong may serve as a valuable genetic resource for breeding programs targeting climate resilience. Furthermore, native chicken breeds from Thailand and other countries—such as Shee and Pradu Hang Dum Thai native chickens [28,43], native chickens in Cameroon [44], and Beijing You chickens [45]—have also been evaluated under heat stress, revealing similar challenges in balancing growth performance with adaptability. For instance, Thai native chickens possess moderate growth potential but demonstrate resilience under heat and low-input conditions [28,43]. Similarly, native chickens in Cameroon, while exhibiting slower growth rates, are recognized for their robustness and heat tolerance in tropical climates [44]. Beijing You chickens display less impact from heat stress than Guang Ming chickens, maintaining growth performance and demonstrating better adaptability due to superior homeostasis regulation mechanisms, as indicated by reduced differentially expressed genes and physiological changes under heat stress [45]. Incorporating such international comparisons provides a broad context for interpreting the significance of Thai synthetic lines and reinforces their value in global genetic resource conservation and climate-resilient breeding programs.

Heritability estimates (h^2^) provided essential insights into the genetic potential for improving growth traits through selection in native chicken populations. In the present study, the h^2^ values for BW, ADG, and BrC across the four Thai native synthetic chicken lines revealed notable variation in genetic control across age stages and lines. These findings were particularly relevant for breeders aiming to enhance productivity while maintaining adaptability to tropical environments. Heritability for BW0 ranged from moderate to high (0.36–0.45), with Kaimook e-san exhibiting the highest estimate. This result indicated that early growth was predominantly governed by additive genetic effects, rendering it a promising selection target in breeding programs [46,47]. However, the h^2^ values for BW declined progressively with age, dropping to 0.26–0.31 by 14 weeks (BW14). This decline aligned with previous findings and was likely owing to increasing environmental influences and physiological maturity, which can obscure the expression of genetic variance in later growth stages [48,49]. However, some previous studies have reported contrasting results, indicating that heritability tends to increase with the age of the chickens [50,51]. A similar pattern was observed for ADG traits. The highest heritability was recorded during the 0–4 week interval, reflecting stronger genetic control during early post-hatch growth. By contrast, heritability for ADG12–14 was notably lower, suggesting greater environmental sensitivity and potential genotype-by-environment interactions during this late growth period, which coincides with heightened vulnerability to heat stress in tropical systems [28,52]. Despite the decline in heritability, the additive genetic variance for most traits—particularly in Kaimook e-san—remained stable, indicating that genetic selection could still yield improvement. For BrC, moderate h^2^ values during early growth stages (0.24–0.33) suggested potential for genetic improvement in muscling traits, which are closely related to carcass yield. Similarly to heritability for BW and ADG, heritability for BrC declined at 14 weeks, possibly because of the increasing influence of environmental stress and physiological load on carcass development.

Understanding genetic correlations between traits is essential in poultry breeding, particularly when improvement in one trait could inadvertently compromise another. In the present study, the genetic correlations revealed meaningful interactions between growth traits and heat tolerance across the four synthetic Thai native chicken lines. In Kaimook e-san, the strongest negative genetic correlations were observed, particularly between heat tolerance and all growth traits, including BW, ADG, and BrC. This result indicated that selection for rapid growth and enhanced muscle deposition in this line was genetically associated with reduced heat tolerance. Such antagonistic pleiotropy aligns with previous findings in tropical poultry populations, where fast-growing birds demonstrated physiological limitations under heat stress due to elevated metabolic heat production and reduced capacity for thermal dissipation [24,53]. Soi Pet exhibited a similar pattern, although the correlations were slightly weaker (ranging from −0.65 to −0.80), suggesting a comparable but less intense genetic constraint. These findings imply that while Soi Pet may be selected for improved growth performance, caution must be exercised to prevent excessive losses in heat tolerance. This inverse relationship between growth performance and heat tolerance may reflect, in part, differences in metabolic load; however, further physiological evidence—such as measures of feed intake, thermoregulatory capacity, or stress protein expression—is needed to substantiate this potential causal link.

The moderate negative correlation between BrC14 and heat tolerance (r = −0.70) further suggested that selection for carcass traits could impair thermal resilience, consistent with the findings by Schmidt et al. [54]. Contrastingly, Soi Nin and Kaen Thong showed weaker or near-neutral genetic correlations (ranging from −0.30 to −0.70), particularly in Kaen Thong, where the relationship between growth traits and heat tolerance was minimal. This relative genetic independence offered greater flexibility for simultaneous selection, positioning Kaen Thong as a promising genetic resource for achieving balanced improvement in productivity and adaptability. These results support the growing consensus that native and synthetic chicken lines harbor valuable alleles for combining growth potential with environmental resilience, an increasingly important objective in the face of climate change [14,55,56].

We assessed the genetic progress of growth traits and heat tolerance across four Thai native synthetic chicken lines over seven generations under tropical environmental conditions. The results demonstrated consistent genetic improvement in key growth parameters, including BW, ADG, and BrC, particularly in Kaimook e-san. However, this improvement was accompanied by a concurrent genetic decline in heat tolerance, reinforcing the presence of an antagonistic genetic relationship between productivity and heat tolerance in poultry. Kaimook e-san achieved the highest genetic gains across all growth traits, with a ΔG of 14.92 g/gen for BW14 and 2.51 g/day/gen for ADG12–14. These results suggest strong additive genetic responsiveness to directional selection for late-stage growth. Previous studies have similarly reported that long-term selection for enhanced growth in poultry can yield substantial genetic gains in BW and muscling traits [57,58,59]. However, as observed in the present study, such improvements often lead to increased physiological stress and reduced adaptability, particularly under hot and humid environments [1,60,61].

Notably, heat tolerance EBVs demonstrated a declining trend in all lines, with the steepest reductions found in Kaimook e-san (−1.49 g/gen for BW14-associated heat tolerance). These findings support earlier reports indicating that selection for fast growth elevates metabolic heat production, compromising the birds’ ability to dissipate heat efficiently and maintain homeostasis under thermal stress [8,24]. The genetic decline in heat tolerance for ADG12–14 and BrC14 further confirmed this antagonism. However, the magnitude of decline associated with BrC was comparatively moderate, suggesting that selection for carcass traits may exert a less pronounced thermal burden than selection for metabolic traits. Interestingly, among the lines, Kaen Thong consistently exhibited the lowest genetic gains across all growth traits and the smallest decline in heat tolerance. This pattern could reflect a more balanced genetic architecture or lower selection intensity in this line. The genetic stability in Kaen Thong may be advantageous in breeding programs focused on environmental adaptability and long-term sustainability [62].

The cumulative evidence highlights the importance of integrating heat tolerance into selection indices, particularly for poultry populations raised in tropical and subtropical regions. While selection for growth performance remains economically important, neglecting heat stress resilience could undermine productivity gains due to increased mortality, reduced feed intake, and compromised immune responses [26]. Future breeding strategies should consider multi-trait selection approaches that simultaneously enhance growth and maintain physiological stability under climate stressors.

One of the most critical findings is the antagonistic relationship between growth performance and heat tolerance. Heat tolerance EBVs for BW14 declined significantly in the highest-growing lines, particularly in Kaimook e-san (−1.49 g/gen) and Soi Pet (−1.26 g/gen). Similar negative trends were observed in heat tolerance for ADG and BrC (Figure 3D–F). These results confirm that selection for rapid growth traits compromises heat resilience, likely due to physiological and metabolic trade-offs. Chickens selected for fast growth typically have higher metabolic rates, larger body mass, and increased heat production, making them more susceptible to thermal stress [23,24]. This is particularly critical in tropical environments, where ambient temperatures frequently exceed the thermoneutral zone of poultry, reducing feed intake, growth, and survivability [1]. Moreover, the crossing of curves among different strains (e.g., A, B, and D) suggests the presence of genotype-by-environment (G×E) interactions, particularly in response to varying levels of heat stress. This indicates that the relative performance of the strains is not consistent across environmental conditions—some strains may perform better under mild conditions, while others exhibit greater resilience under severe heat stress. Such interactions highlight the importance of considering environmental sensitivity in selection programs. The revised discussion now addresses this point accordingly.

These findings underscore the necessity of balanced poultry breeding selection in tropical climates. While selective breeding effectively enhanced growth traits, it inadvertently compromised the birds’ ability to cope with thermal stress, particularly in the later growth phases. Without the integration of heat tolerance traits into selection indices, breeding programs may inadvertently increase vulnerability to heat stress, particularly under current and future climate scenarios. As such, breeding strategies must transition from single-trait to multi-trait selection that balances productivity with adaptability, where index selection or genomic selection integrates growth, muscling, and resilience traits. Moreover, we acknowledge that other indicators—such as survivability, feed intake, and core thermoregulatory responses (e.g., rectal temperature, respiration rate)—are critical to comprehensively evaluating heat stress resilience. However, due to limitations in data availability and consistency across the population, these traits were not included in the present analysis. Future research should aim to integrate these physiological and performance-based parameters to provide a more holistic understanding of adaptive responses in tropical production systems.

## 5. Conclusions

In this study, we evaluated the phenotypic performance, genetic parameters, and genetic trends of four Thai native synthetic chicken lines—Kaimook e-san, Soi Pet, Soi Nin, and Kaen Thong—focusing on growth traits and heat tolerance under tropical conditions. Among the four chicken lines, Kaimook e-san consistently exhibited the highest genetic gains (ΔG) across all growth-related traits, particularly for BW14, ADG12–14, and BrC14. These findings reflected the strong genetic responsiveness of Kaimook e-san to directional selection aimed at improving productivity traits. However, this genetic progress was accompanied by a concurrent decline in heat tolerance, highlighting the antagonistic genetic relationship between growth and heat tolerance. Therefore, future selection strategies should aim to incorporate heat resilience traits alongside growth performance to ensure long-term adaptability and sustainability under climate-stressed production environments.

## Figures and Tables

**Table 1:** Estimated variance components and heritability values (standard error; SE) of BW, ADG, and BrC in four Thai native synthetic chicken lines.

Synthetic Chicken Lines	Kaimook e-san			Soi Pet		
Variances/Heritability	Va	Ve	h^2^ (SE)	Va	Ve	h^2^ (SE)
Traits						
BW0	8.77	10.89	0.45 (0.05)	7.22	11.02	0.40 (0.05)
BW4	1812	2835	0.39 (0.05)	1715	2763	0.38 (0.05)
BW8	3782	7687	0.33 (0.05)	3544	7511	0.32 (0.04)
BW12	12,602	28,364	0.31 (0.04)	11,999	27,421	0.30 (0.04)
BW14	17,277	38,752	0.31 (0.04)	15,600	35,552	0.30 (0.04)
ADG0–4	1.02	1.98	0.34 (0.03)	0.89	2.00	0.31 (0.04)
ADG4–8	2.18	4.59	0.32 (0.03)	1.77	4.22	0.30 (0.04)
ADG8–12	2.85	6.09	0.32 (0.03)	2.55	6.52	0.28 (0.04)
ADG12–14	3.30	7.22	0.31 (0.03)	3.01	7.99	0.27 (0.03)
BrC8	0.60	1.22	0.33 (0.03)	0.54	1.32	0.29 (0.03)
BrC12	0.85	1.90	0.31 (0.03)	0.75	2.01	0.27 (0.03)
BrC14	1.02	2.66	0.28 (0.03)	0.84	2.45	0.26 (0.03)
Synthetic chicken lines Variances/Heritability	Soi Nin			Kaen Thong		
	Va	Ve	h^2^ (SE)	Va	Ve	h^2^ (SE)
Traits						
BW0	7.01	11.25	0.38 (0.05)	6.87	12.01	0.36 (0.05)
BW4	1599	2944	0.35 (0.05)	1423	3012	0.32 (0.04)
BW8	3457	7422	0.32 (0.04)	3002	7315	0.29 (0.04)
BW12	11,524	26,782	0.30 (0.04)	9625	25,676	0.27 (0.03)
BW14	14,100	34,256	0.29 (0.04)	11,562	33,253	0.26 (0.03)
ADG0–4	0.86	1.99	0.30 (0.04)	0.75	1.89	0.28 (0.03)
ADG4–8	1.68	4.22	0.28 (0.03)	1.42	4.01	0.26 (0.03)
ADG8–12	2.35	6.58	0.26 (0.03)	1.86	5.89	0.24 (0.03)
ADG12–14	2.88	8.56	0.25 (0.03)	2.12	7.53	0.22 (0.03)
BrC8	0.50	1.45	0.26 (0.03)	0.45	1.40	0.24 (0.03)
BrC12	0.65	1.85	0.26 (0.03)	0.52	1.77	0.23 (0.03)
BrC14	0.79	2.35	0.25 (0.03)	0.64	2.22	0.22 (0.03)

BW0, BW4, BW8, BW12, and BW14 = hatching weight, body weight at 4, 8, 12, and 14 weeks of age, respectively (g); ADG0–4, ADG4–8, ADG8–12, and ADG12–14 = average daily gain at 0–4, 4–8, 8–12, and 12–14 weeks of age, respectively (g/day); BrC8, BrC12, and BrC14 = breast circumference at 8, 12, and 14 weeks of age, respectively (cm); Va = additive genetic variances; Ve = residual variances; $h^{2}=$ heritability.

**Table 2:** Average genetic progress (ΔG) per generation for BW, ADG, BrC, and heat tolerance in four Thai native synthetic chicken lines.

Chicken Lines	Kaimook e-san		Soi Pet		Soi Nin		Kaen Thong	
BW traits (g/gen)	BW	Heat tolerance	BW	Heat tolerance	BW	Heat tolerance	BW	Heat tolerance
BW0	0.15	−0.02	0.13	−0.01	0.10	−0.01	0.05	−0.00
BW4	6.23	−0.62	5.99	−0.58	5.65	−0.40	5.02	−0.35
BW8	9.10	−0.91	8.44	−0.78	8.01	−0.66	7.45	−0.57
BW12	12.12	−1.21	10.89	−1.07	9.42	−0.76	7.85	−0.69
BW14	14.92	−1.49	13.03	−1.26	11.18	−0.96	8.10	−0.85
ADG traits (g/gen)	ADG	Heat tolerance	ADG	Heat tolerance	ADG	Heat tolerance	ADG	Heat tolerance
ADG0–4	0.54	−0.06	0.38	−0.04	0.32	−0.03	0.25	−0.02
ADG4–8	1.38	−0.17	1.21	−0.12	1.14	−0.09	1.01	−0.07
ADG8–12	2.04	−0.24	1.88	−0.19	1.65	−0.17	1.42	−0.13
ADG12–14	2.51	−0.30	2.29	−0.26	2.00	−0.22	1.94	−0.17
BrC traits (cm/gen)	BrC	Heat tolerance	BrC	Heat tolerance	BrC	Heat tolerance	BrC	Heat tolerance
BrC8	0.25	−0.05	0.18	−0.05	0.14	−0.04	0.12	−0.03
BrC12	0.55	−0.10	0.46	−0.09	0.40	−0.07	0.36	−0.05
BrC14	0.92	−0.13	0.77	−0.12	0.68	−0.10	0.60	−0.08

Heat tolerance as the animal-specific regression slope of estimated breeding values (EBVs) on THI, representing the animal’s genetic sensitivity to heat stress.

## Data Availability

The original contributions presented in the study are included in the article. Further inquiries can be directed to the corresponding author.
